# Interplay of lamin A and lamin B LADs on the radial positioning of chromatin

**DOI:** 10.1080/19491034.2019.1570810

**Published:** 2019-01-20

**Authors:** Frida Forsberg, Annaël Brunet, Tharvesh M. Liyakat Ali, Philippe Collas

**Affiliations:** a Department of Molecular Medicine, Institute of Basic Medical Sciences, Faculty of Medicine, University of Oslo, Oslo, Norway; b Norwegian Center for Stem Cell Research, Department of Immunology and Transfusion Medicine, Oslo University Hospital, Oslo, Norway

**Keywords:** Chromatin, LAD, nuclear lamins, genome conformation, 3D genome

## Abstract

Immunosuppressive drugs such as cyclosporin A (CsA) can elicit hepatotoxicity by affecting gene expression. Here, we address the link between CsA and large-scale chromatin organization in HepG2 hepatocarcinoma cells. We show the existence of lamina-associated domains (LADs) interacting with lamin A, lamin B, or both. These ‘A-B’, ‘A-only’ and ‘B-only’ LADs display distinct fates after CsA treatment: A-B LADs remain constitutive or lose A, A-only LADs mainly lose A or switch to B, and B-only LADs remain B-only or acquire A. LAD rearrangement is overall uncoupled from changes in gene expression. Three-dimensional (3D) genome modeling predicts changes in radial positioning of LADs as LADs switch identities, which are corroborated by fluorescence *in situ* hybridization. Our results reveal interplay between A- and B-type lamins on radial locus positioning, suggesting complementary contributions to large-scale genome architecture. The data also unveil a hitherto unsuspected impact of cytotoxic drugs on genome conformation.**Abbreviations:** ChIP-seq: chromatin immunoprecipitation sequencing; CsA: cyclosporin A; FISH; fluorescence *in situ* hybridization; ICMT: isoprenylcysteine methyltransferase; LAD: lamina-associated domain; TAD: topologically-associated domain

## Introduction

Drug-induced hepatotoxicity, a common cause of clinical trial failure, has led to the use of cellular models such as HepG2 hepatocarcinoma cells for drug testing [1,2]. At micromolar concentrations, the steatotic drug cyclosporin A (CsA) inhibits several signaling pathways in HepG2 cells [3], resulting in metabolic alterations [1,3,4]. Interestingly, CsA can also act by modulating the binding of transcription factors to chromatin [3], suggesting an impact on genome organization.

The mammalian genome is organized into compartments of active and inactive chromatin [5] and within these, regions of high-frequency chromosomal interactions termed topologically associated domains (TADs) [6,7]. The genome is also radially organized, with lamina-associated domains (LADs) anchoring chromatin to the nuclear lamina, at the nuclear periphery [8], a meshwork of lamins A/C (from here on, ‘lamin A’) and B [9]. As such, lamin-chromatin interactions play an important role in the radial (center – periphery) positioning of loci in the nucleus [10]. LADs are typically 0.1–10 megabases (Mb), gene-poor, enriched in heterochromatin and display low gene activity [8,11]. While lamins A and B are localized at the nuclear lamina, a nucleoplasmic pool of lamin A also interacts with euchromatic regions [12–16]. Intriguingly, a minor fraction of lamin B1 has also been found in the nuclear interior also in association with euchromatin [17]. These observations suggest that alterations in lamin-genome interactions may impact genome organization at the nuclear periphery and in the nuclear interior.

Lamin-chromatin interactions can be altered during differentiation [14,15,18–20], indicating that some of these interactions are dynamic. Moreover, lamins A and B can form LADs that overlap but can also be distinct [12,13], suggesting redundant but also complementary roles of A- and B-type lamins in the modulation of radial genome conformation [21,22].

In light of these observations, to address the relationship between CsA exposure and genome organization, we examined here the effect of CsA on the association of chromatin with nuclear lamina. We report an impact of CsA on the dynamics of interactions of lamins A and B with the genome. Such interactions can be gained, lost or interchanged in a manner uncoupled from gene expression changes. They also correlate with distinct patterns of radial repositioning of loci predicted from 3D genome models and confirmed by fluorescence *in situ* hybridization (FISH). The data suggest an A- and B-type lamin interplay in radial genome conformation and reveal unsuspected effects of cytotoxic compounds such as CsA on nuclear organization.

## Results

### CsA elicits pre-lamin A accumulation

**Figure 1.**
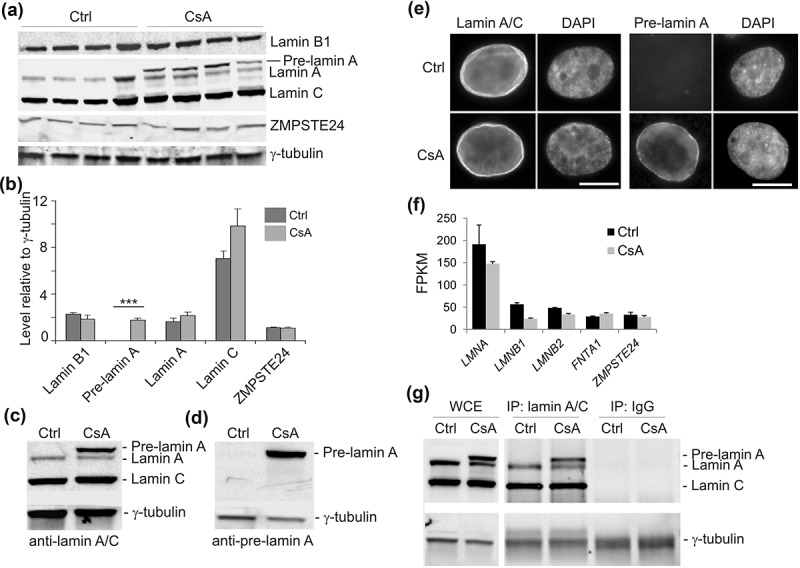
Cyclosporin A elicits pre-lamin A accumulation in HepG2 cells. (a) Western blot analysis of nuclear lamins and ZMPSTE24 in control (Ctrl) and HepG2 cells treated with 10 µM CsA for 72 h. γ-tubulin was used as loading control; data from 4 experiments. Anti-lamin A/C antibody used was a characterized rabbit antibody [44]. (b) Quantification of the blot shown in (a), relative to γ-tubulin; mean ± SD; ***P = 6.0 × 10^−5^, paired t-tests relative to Ctrl. (c) Western blot of lamin A/C using the Santa-Cruz sc7292x anti-lamin A/C antibody used for ChIP. (d) Confirmation of pre-lamin A induction using a pre-lamin A antibody (Santa-Cruz sc6214). (e) Immunofluorescence labeling of lamin A/C (sc7292x) and pre-lamin A (sc6214 antibody). DNA was stained with DAPI. Bars, 10 µm. (f) Expression of lamin genes and *ZMPSTE24* in control and CsA-treated cells (mean ± SD FPKM from duplicate RNA-seq data). *FNTA1* was used as unaltered expression control. (g) Western blot of lamin A/C in whole cell extract (WCE) and after immunoprecipitation (IP) of lamin A/C (sc7292x) or IP with an irrelevant IgG, from control and CsA-treated cells under ChIP conditions. Detection was with the rabbit anti-lamin A/C antibody.

Before investigating changes in genome organization that might be elicited by CsA, we determined whether CsA altered levels of nuclear lamins. We used 10 µM CsA, a concentration in the range of doses used in hepatotoxicity assays [4,23]. This dose is sub-cytotoxic over the 72 h period considered here, avoiding necrotic or apoptotic drawbacks [3]. Western blot analysis shows that exposure of HepG2 cells to CsA did not alter levels of lamins A/C and B1; however CsA elicited consistent and significant pre-lamin A accumulation (P = 6 × 10^−5^], paired t-tests relative to controls; Figure 1(a,b)). This was verified using another lamin A/C antibody (Santa-Cruz sc7292x) and an antibody against pre-lamin A (Santa-Cruz sc6214) (Figure 1(c,d)). Immunofluorescence labeling confirmed the upregulation and localization of pre-lamin A at the nuclear periphery (Figure 1(e)). We also generated RNA-sequencing (RNA-seq) data for control and CsA-treated cells, and show that CsA did not alter *LMNA* or *LMNB1* transcript levels (Figure 1(f); Supplementary Table S1).

Importantly, CsA does not affect protein or transcript levels of ZMPSTE24 (Figure 1(a,f)), the protease involved in lamin A maturation [9], suggesting that processes other than altered ZMPSTE24 levels interfere with lamin A maturation upon CsA exposure. This finding is consistent with the fact that ablation of ZMPSTE24 in mice results in complete inhibition of pre-lamin A maturation [24]. Our findings, rather, are reminiscent of partial pre-lamin A processing observed after depletion or inhibition of isoprenylcysteine carboxymethylation [24]. We cannot at present exclude that this pre-lamin A accumulation results from a senescence phenotype or cellular stress elicited by CsA [1,2,23,25]. Accumulation of pre-lamin A at the nuclear envelope however suggests that interactions of chromatin with the nuclear lamina could be altered.

### Lamin A association with lamin B LADs

**Figure 2.**
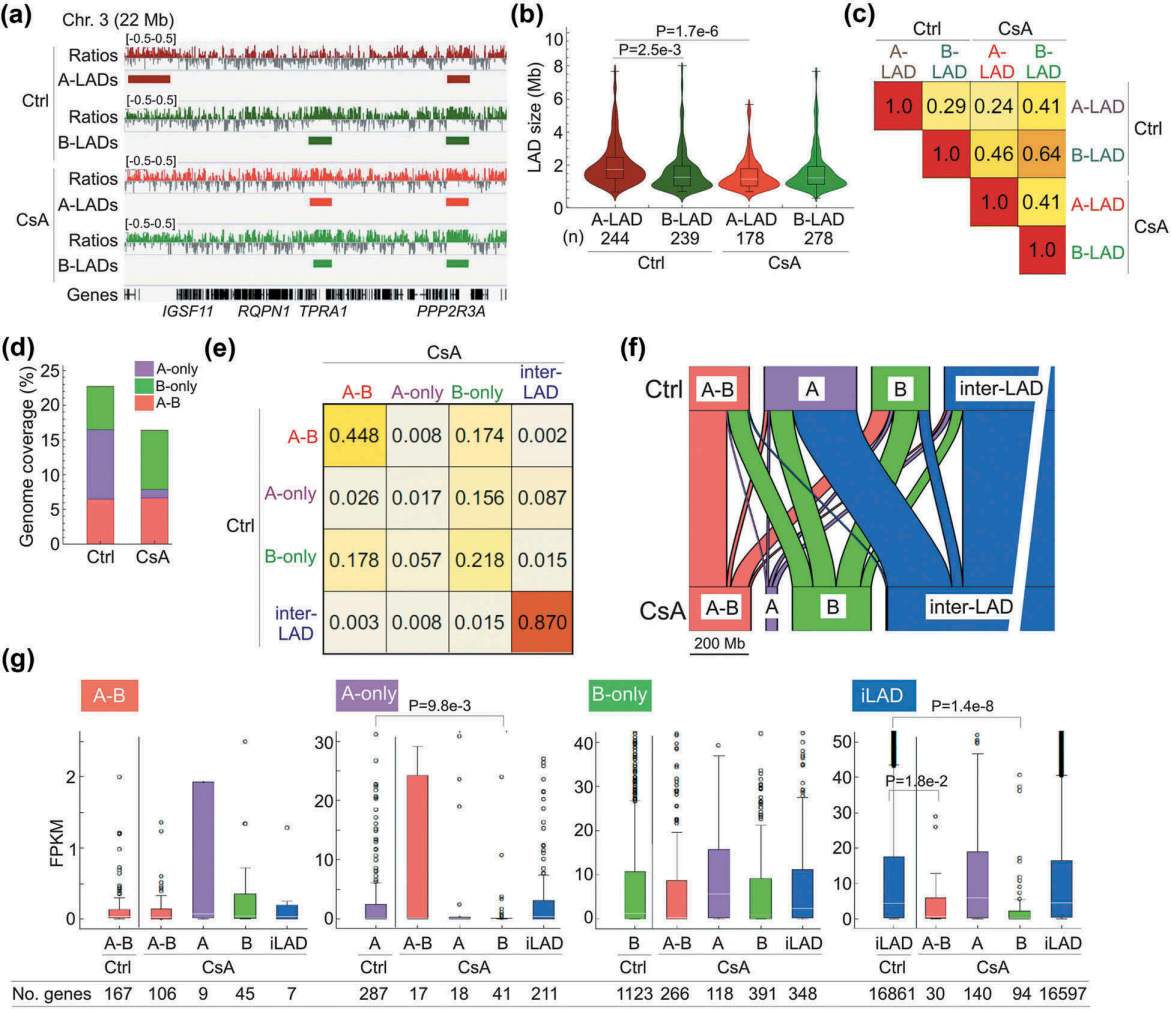
Differential patterns of lamin A and B association with the genome. (a) Browser view of lamin A/C and B1 ChIP-seq profiles of log_2_(ChIP/input) ratios and of mapped A- and B-LADs. (b) LAD size distribution (n, number of LADs; P-value: 2-way ANOVA followed by Tukey’s HSD posthoc test). (c) Jaccard indices of genome coverage by A- and B-LADs in control and CsA-treated cells. A ratio of 1 indicates a perfect overlap. (d) Relative genome coverage of A-B, A-only and B-only LADs. (e) Jaccard indices of overlap of all LAD classes in control and CsA-treated cells. (f) Alluvial representation of the outcome of A-B LADs, A-only LADs, B-only LADs and inter-LADs in CsA-treated cells. Width of boxes reflects proportions of each LAD class in the genome; width of lines reflects proportions of LAD classes turning into another. (g) Gene expression levels in each LAD class in control cells (Ctrl) and in their derivative sub-classes after CsA treatment (CsA). FPKMs are from RNA-seq data. Significant P-values (< 0.01; unpaired t-tests) and numbers of genes in each LAD class are shown.

We thus determined whether LADs were remodeled in CsA-treated cells. We mapped lamin B LADs (from here on called ‘B-LADs’) and lamin A LADs (‘A-LADs’) by chromatin immunoprecipitation-sequencing (ChIP-seq) of lamin B1 and lamin A/C, respectively. Of note, the anti-lamin A/C ChIP antibody (Santa-Cruz sc7292x) immunoprecipitated not only lamin A/C but also pre-lamin A (Figure 1(g)), ruling out a distinction between chromatin binding to pre-lamin A and matured lamin A/C. We respectively identify in control and CsA-treated cells 244 and 178 A-LADs, and 239 and 278 B-LADs, each ranging from ~0.5 to ~15 megabases (Mb) (Figure 2(a,b); Supplementary Table S2). While size and genome coverage of B-LADs are not altered by CsA, number and size of A-LADs are lower, resulting in a 50% reduction in A-LAD coverage (Figure 2(b); Supplementary Table S2). This reduced association of chromatin with lamin A is likely not due to less efficient lamin A/C ChIP in CsA-treated cells because Western blot analysis reveals similar amounts of immunoprecipitated lamins A and C in these cells and in controls (Figure 1(g)). Alterations observed in our LAD analyses suggest marked changes in the distribution of A- and B-LADs.

We therefore examined the overlap of A- and B-LADs in control and CsA-treated cells by computing Jaccard indices (Ji) of their genome coverage (Figure 2(c)). We find a low overlap between A-LADs and B-LADs in control cells (Ji 0.29), reflecting distinct A- and B-LAD subpopulations. Moreover, whereas B-LADs are well conserved in CsA-treated cells (Ji 0.64), A-LADs show little overlap (Ji 0.24), reflecting a redistribution of these LADs (in addition to the loss reported above). Interestingly however, A-LADs after CsA treatment coincide with B-LADs in control cells (Ji 0.46; Figure 2(a,c)), indicating a significant overlap of *de novo* A-LADs with pre-existing B-LADs.

### Distinct behaviors of lamin A and B association with chromatin

To further investigate the relationship between A-LADs and B-LADs, we divided these into LAD classes associated with lamin A only, lamin B only and both lamins A and B (‘A-only’, ‘B-only’ and ‘A-B’ LADs) and analyzed the properties of these LADs in control and CsA-treated cells (Supplementary Table S3). We find that (i) genome coverage by B-only LADs slightly increases (Figure 2(d)) due to an increase in LAD number (Supplementary Figure S1(a)); however, overlap of these B-only LADs is low (Ji 0.218; Figure 2(e)), indicating a genomic redistribution after CsA treatment. (ii) In contrast, A-only LADs show reduced coverage resulting from lower numbers and size (Figure 2(d); Supplementary Figure S1(a)), and no overlap with LADs in controls (Ji 0.017; Figure 2(e)). (iii) A-B LADs are the most conserved, although variation in their position also occurs (Figure 2(d,e); Supplementary Figure S1(a)). We infer from these data a redistribution of A-only and B-only LADs after CsA treatment, while A-B LADs are the most conserved.

We further examined the fate of these LADs using an alluvial graph representation of the extent to which A-only, B-only and A-B LADs remained unaltered or turned into another LAD class with CsA. Figure 2(f) shows that (i) A-B LADs mostly remain ‘A-B’ while a smaller fraction loses lamin A to become B-only. (ii) Strikingly, most A-only LADs lose lamin A and become inter-LADs while the remaining switch to B-only or become A-B. (iii) B-only LADs mainly remain B-only, while ~30% turn into A-B, demonstrating the *de novo* acquisition of lamin A on B LADs inferred from Jaccard indices (Figure 2(e,f); Supplementary Table S4). (iv) *De novo* A-only and B-only LADs appear in inter-LAD regions, albeit in minor proportions (Figure 2(f)). These results argue that association of lamin A with the genome is more dynamic than that of lamin B in this experimental system. They also highlight a rearrangement of lamin-genome interactions through lamin loss, lamin exchange or acquisition of lamin A on regions interacting with lamin B.

### Gene expression changes associated with LAD dynamics

We next determined whether LAD dynamics was associated with changes in gene expression. Genes in A-B, A-only and B-only LADs are not significantly differently expressed after CsA treatment (Supplementary Figure S1(b), P > 0.01, unpaired t-tests) although trends are detectable. We thus asked whether genes in each LAD class in control cells showed altered expression as a function of LAD class fate after CsA treatment. Due to the low number of genes in LAD classes of CsA-treated cells, many LAD class changes were not associated with significant expression changes (Figure 2(g)). However, genes in A-only LADs switching to B-only were downregulated (P = 9.8 × 10^−3^; unpaired t-tests; Figure 2(g), ‘A-only’). Acquisition of A-B or B-only LADs from inter-LADs was also accompanied by downregulation of gene expression (P = 0.018 and 1.4 × 10^–8^ respectively, unpaired t-tests; Figure 2(g), ‘iLAD’). Thus, a gain of lamin B interaction, including a switch from lamin A to B, correlates with expression downregulation in these LADs. In contrast, gain or loss of lamin A, or loss of lamin B, is uncoupled from gene expression changes (Figure 2(g)). We conclude that in line with earlier findings [26], LAD dynamics is overall unlikely to be driven by transcriptional changes.

### Prediction of radial repositioning of loci by CsA

**Figure 3.**
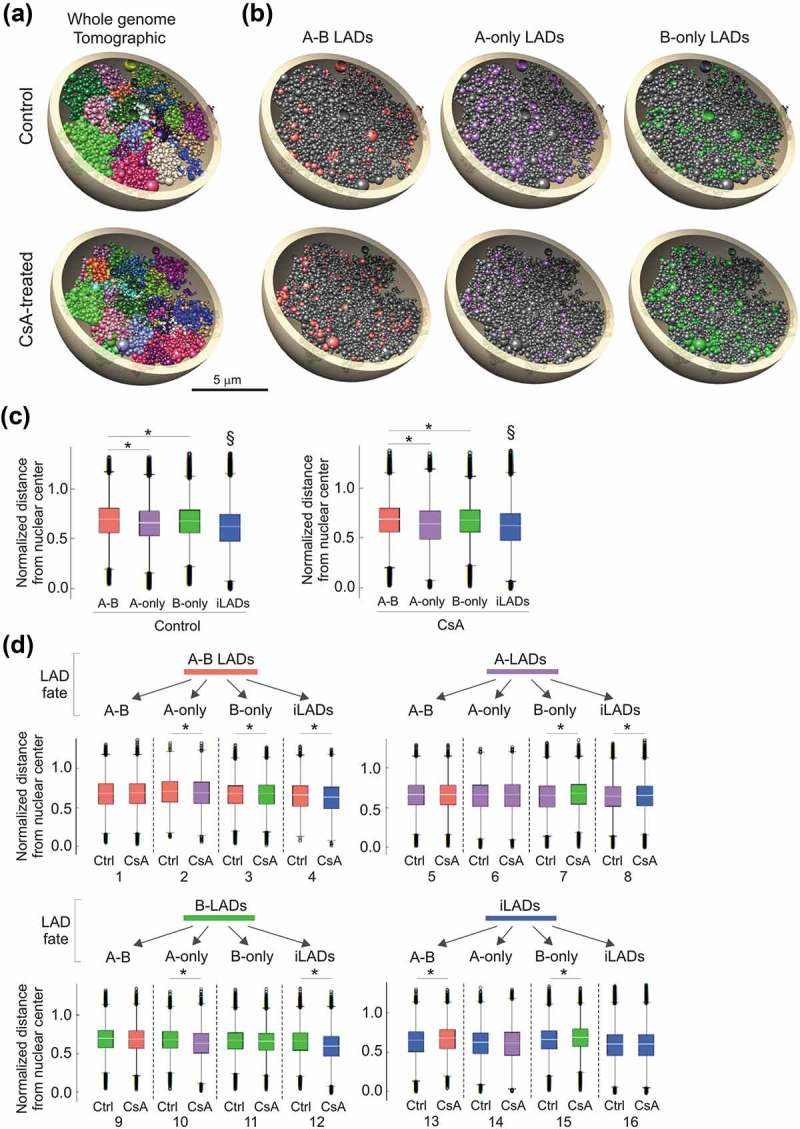
Structural 3D genome modeling reveals changes in radial LAD positioning related to LAD fate. (a) Chrom3D models of the HepG2 genome built from Hi-C and lamin B1 ChIP-seq data used as positional constraints for TADs and LADs. Tomographic views show individual chromosomes (differentially colored) modeled as beads on a string, each bead representing a TAD identified in the Hi-C data. One example structure out of 800 generated for control and CsA-treated cells is shown. (b) Tomographic views of the structures shown in (a) showing A-B, A-only and B-only LADs in control and CsA-treated cells. Beads (TADs) may harbor more than one LAD class due to their size. (c) Normalized LAD distance from the nuclear center across 800 3D structures of control and CsA-treated cells, for each LAD class and for all remaining TADs (inter-LADs). ^§^P < 2.2 × 10^−16^ compared to each LAD class; unpaired t-tests; *P < 2.2 × 10^−16^; unpaired t-tests. These distances were calculated as absolute distances divided by the radius of the modeled nucleus (5 µm). (d) Normalized LAD distance from nuclear center for each LAD class and all remaining TADs (iLADs);*P < 2.2 × 10^−16^, unpaired t-tests. LAD fate diagram (top) reflects the transition from one LAD class to another (see Figure 2F). Compared sample pairs (see main text) are numbered at the bottom.

To assess whether a gain or loss of LAD or a change in LAD identity would coincide with changes in the radial positioning of these domains, we generated 3-dimensional (3D) models of the HepG2 genome and analyzed properties of the models. We used Chrom3D, a 3D genome modeling platform that integrates TAD-TAD interactions from Hi-C data, and genome interactions with the nuclear periphery from lamin ChIP-seq data [27,28]. Our input data were Hi-C data for HepG2 (ENCODE, NCBI GEO accession GSE105381, sample GSM2825569) and lamin B1 ChIP-seq data for control and CsA-treated cells. We generated 800 Chrom3D models for each condition, mapped A-B, A-only and B-only LADs onto the models and computed their normalized distances to the nuclear center to estimate their radial position. The structures recapitulate chromosome territories within which LADs can be highlighted (Figure 3(a,b)). Analysis of the models shows that A-B, A-only and B-only LADs are as expected more peripheral than inter-LADs, and between LAD classes, A-only LADs are more centrally placed than A-B or B-only LADs (P < 2.2 × 10^−16^, unpaired t-tests; Figure 3(c)).

We next estimated the radial repositioning of each LAD class according to their fate in CsA-treated cells (Figure 3(d)). Loss of lamin association altogether correlates with repositioning of loci towards the nuclear center (Figure 3(d), comparison pair 4, 8, 12; P < 2.2 × 10^−16^). Moreover, retention of a LAD class does not result in significant repositioning. Significantly however, loss of lamin B from an A-B LAD (pair 2) or a switch from a B-only to an A-only LAD (pair 10) correlates with repositioning towards the center (P < 2.2 × 10^−16^). In contrast, gain of lamin B resulting from an A-only to B-only switch (pair 7), a *de novo* gain of A-B (pair 13) or a *de novo* gain of B-only (pair 15), correlates with repositioning towards the periphery (P < 2.2 × 10^−16^). Lastly, gain of lamin A does not correlate with significant repositioning (pair 9, 14); however the impact of lamin A loss on radial placement depends on whether lamin B is bound in the region (pair 3, 4, 7, 8; P < 2.2 × 10^−16^). Analysis of 3D genome models therefore predicts a dominant impact of lamin B over lamin A on radial positioning of the genome.

### FISH analysis validates predictions on locus repositioning from 3D models

**Figure 4.**
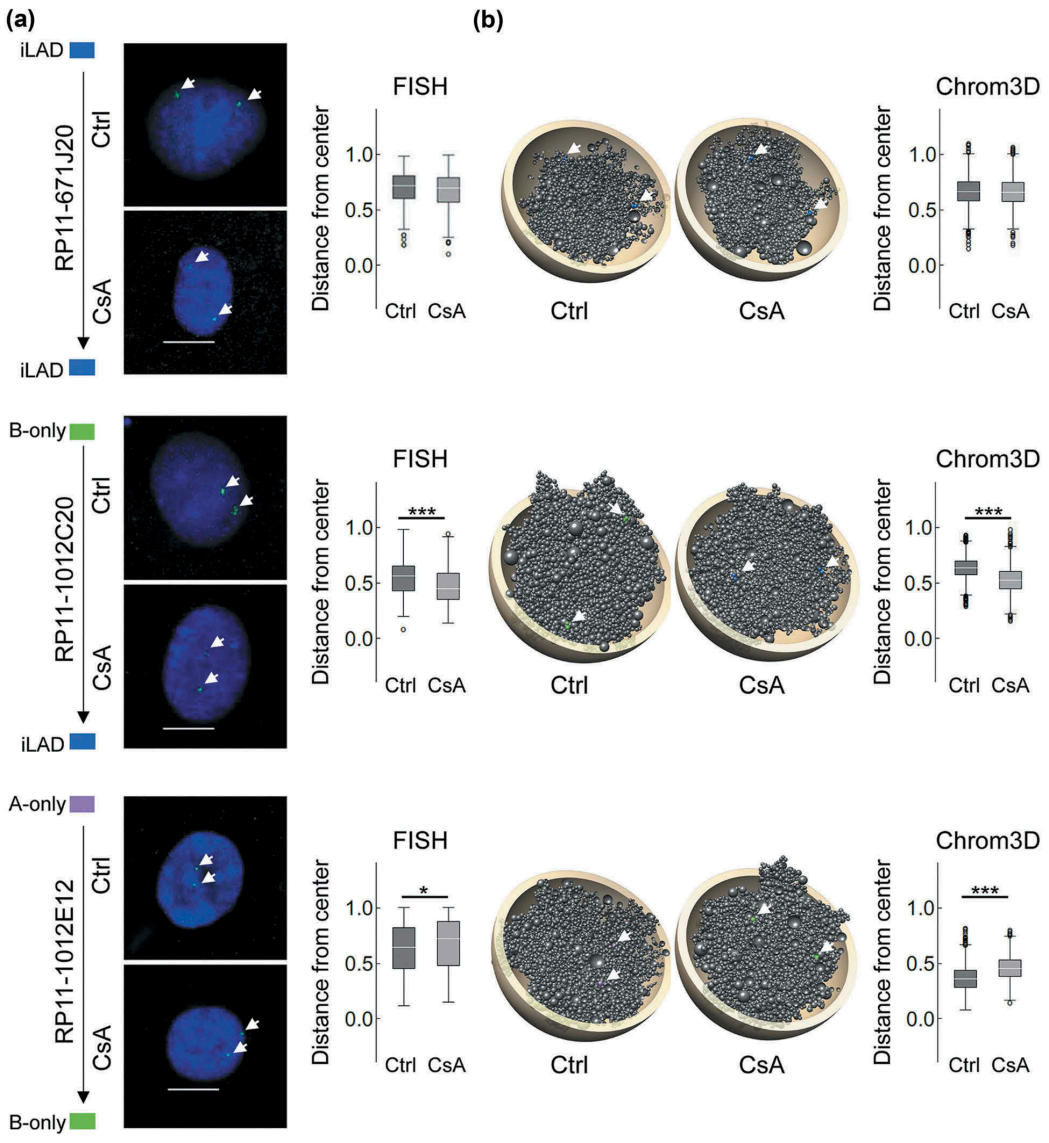
Chrom3D and FISH analysis of radial repositioning of loci after CsA treatment. (a) FISH analysis of locus positioning (arrows) in indicated LADs in control and CsA-treated cells. Representative FISH images are shown, with normalized probe distance from the nuclear center (0 = center; 1 = periphery defined as the border of DAPI staining; n = 100 nuclei analyzed per probe per condition; bars, 10 µm), (b) Chrom3D models showing FISH probe positions (arrows), and normalized probe distance from the nuclear center measured in 800 models. Probe number is shown. P-values: FISH: *P = 0.006, ***P = 1.4 × 10^−5^; unpaired t-tests; Chrom3D: ***P < 2.2 × 10^−16^, unpaired t-tests. See also Supplementary Figure S2.

To independently validate Chrom3D model predictions on locus repositioning, we designed FISH probes to loci found in specific LAD classes in the ChIP-seq data (Supplementary Table S5). We also positioned in Chrom3D models the loci targeted by each FISH probe and measured their distance from the nucleus center (Figure 4(a,b); Supplementary Figure S2(a,b)). FISH analysis shows that maintenance of an inter-LAD does not alter radial positioning (Figure 4(a)), consistent with modeling predictions of FISH probe position (Figure 4(b)) and with genome-wide 3D models (see Figure 3(d), pair 16). Similarly, we observe no repositioning after formation of A-B LADs from A- or B-only LADs, or after loss of an A-only LAD (Supplementary Figure S2). However, loss of a B-only LAD reveals locus-dependent repositioning towards the nuclear center (Figure 4; Supplementary Figure S2). Lastly, a switch from an A-only to B-only LAD correlates with peripheral locus repositioning (P = 0.006, t-tests; Figure 4(a)), again validating model predictions (Figure 4(b); P < 2.2 × 10^−16^, t-tests) (Supplementary Figure S2). Thus, FISH data validate predictions from the 3D models and confirm that lamin B association influences the radial distribution of loci more prominently than lamin A.

Pre-lamin A accumulation following CsA exposure (see Figure 1(a)) could influence locus positioning independently of CsA treatment *per se*. To address this, we expressed a Flag-tag wild-type (wt) pre-lamin A construct. Since, as we have shown earlier, wt pre-lamin A is fully processed into mature lamin A^27^, we also expressed a lamin A mutant (L647R) exclusively localized at the nuclear periphery as unprocessed pre- lamin A^27^ by retention of its pre-lamin A farnesyl group [29]. We then determined by immuno-FISH using anti-Flag antibodies the radial positioning of loci localized in an inter-LAD, in a B-only LAD losing B, and in an A-only LAD switching to B (see Figure 4(a)).

The data strikingly reveal no significant repositioning of the three loci in lamin A L647R vs. wt cells (Supplementary Figure S3). Thus, overexpression of pre-lamin A (as lamin A L647R) does not recapitulate locus repositioning detected in CsA-treated cells (which show some pre-lamin A expression). This suggests that relocalization observed after CsA treatment is a CsA-linked phenotype and not a pre-lamin A expression phenotype *per se*. Repositioning is probably independent of pre-lamin A interaction with chromatin; it is however likely dependent on lamin B, as discussed above.

## Discussion

We report a marked effect of CsA on the association of chromatin with A- and/or B-type lamins. We notably reveal distinct patterns of lamin A and B interaction with chromatin, resulting in variable LAD identities and correlated with a radial repositioning of loci concordant with predictions from 3D genome models. An account of redistribution of lamin A and B (A-B) LADs, A-only and B-only LADs between two cellular states provides biochemical evidence for previous microscopy-based observations of distinct lamin A and B micro-domains [30]. Our results suggest a greater dynamics of lamin A association with chromatin than lamin B, but a dominant influence of lamin B on radial repositioning of loci elicited by a perturbation in cellular state.

Studies of drug hepatotoxicity have revealed an accumulation of pre-lamin A in HepG2 cells exposed to an inhibitor of isoprenylcysteine carboxylmethyltransferases (ICMTs) [31]. Pre-lamin A accumulation in cells treated with CsA is reminiscent of ICMT inhibition or depletion in that a pool of mature lamina is also detected [24]. Thus, proteolytic cleavage of pre-lamin A leading to mature lamin A is partially impaired [9]. ZPMSTE24, the protease implicated in lamin A maturation [9], is not affected by CsA; this is again in line with the detection of mature lamin A after CsA treatment. CsA is not a reported ICMT inhibitor, but could conceivably directly or indirectly affect ICMT activity.

A key question in understanding spatial genome architecture is whether changes in lamin-chromatin interactions reported here occur at the nuclear periphery or in the nuclear interior. Pre-lamin A is detected at the nuclear envelope but could also be nucleoplasmic (if not farnesylated [9]), suggesting that alterations in lamin-chromatin interactions may occur both at the peripheral nuclear lamina and in the nuclear interior. Accordingly, 3D genome modeling and FISH analyses indicate that most changes in LADs and in radial position do not systematically occur *at* the nuclear periphery at the single-cell level. In addition, lamin B-chromatin interactions detected by ChIP from millions of cells do not imply peripheral association of loci in all cells. Our results extend previous findings [27,32,33] and may be explained by dynamic lamin interactions with chromatin [34]. They also support the view that lamin B (in addition to lamin A^12^ [13,15,18,35,36],) may interact with chromatin in the nuclear interior, such as gene-poor perinucleolar heterochromatin [37] or euchromatic regions [17].

Lamins A and B are important regulators of spatial genome organization [21,22,38], and our findings suggest a complementary influence of both lamin types. Lamin association with the genome appears to be more dynamic in regions associated with lamin A than with lamin B, consistent with a view of dynamic A-LADs during adipogenic differentiation [14]. In both experimental systems, lamin B LADs are relatively invariant, while a subset of these also gains lamin A. Nevertheless, the most stable lamin-genome association observed in our data is with A-B LADs. Yet the data suggest that a key contributor to A-B LAD stability is lamin B, which appears to be more stably associated with chromatin than lamin A. Moreover, as our lamin A ChIP precipitates both lamin A and pre-lamin A, we do not at present know whether pre-lamin A accumulation elicited by CsA contributes to LAD dynamics.

Global loss of lamin A results in increased chromatin mobility in the nuclear interior [21]. A loss of A-only LADs in our study may be perceived as a ‘local’ lamin A loss yet bear similar but spatially restricted implications. Thus one may anticipate local gains in chromatin mobility in regions losing lamin A, which is predicted from our modeling data. We therefore raise the hypothesis that not only a global loss of lamin A^21^ but also a more local depletion promotes a restricted conformation change manifested by increased chromatin mobility. It will be relevant to investigate the impact of a local loss of LAD on the biophysical properties of chromatin in the vicinity.

Notwithstanding, repositioning of A-LADs upon loss of lamin A depends on whether lamin B is in the neighborhood. When a lamin B LAD appears *de novo*, we predict and observe locus repositioning towards the periphery. In contrast, in the local absence of lamin B, A-LAD loss correlates with repositioning towards the center. This suggests that lamin B is an important factor in the directional radial organization of chromatin. The surrounding LAD neighborhood could also influence LAD behavior: LAD repositioning may not only depend on the LAD class *per se*, but also on LADs in the vicinity. For instance, absence of repositioning of a B-only LAD towards the nuclear center after losing lamin B, as seen by FISH, could be due to an A-B LAD in the vicinity. Thus, radial genome positioning may be influenced by a combination of both local and medium-range contributions of the linearly nearest region. This idea extends our previous observations that a lamin A neighborhood affects expression of genes in that neighborhood even though they are not bound by lamin A^18^.

These findings raise the question of which factors may regulate the radial positioning of chromatin. Nuclear envelope proteins [38], histone deacetylases [39,40] and histone methyltransferases [41] are central in the radial placement and anchoring of heterochromatin. A FISH-based screen of factors involved in radial placement of a set of genes further shows that locus position is under influence of multiple pathways involving nuclear envelope proteins, centromeric proteins, chromatin remodelers and DNA replication and repair factors [42]. However, as seen in our data, radial repositioning is overall uncoupled from gene activity [42] and thus not driven by transcriptional changes. Nonetheless, radial placement can vary between cell types [42] and may be regulated by cell type-dependent mechanisms. These processes are deregulated under cellular states or disease conditions affecting lamin-chromatin interactions, such as senescence [26] or laminopathies [27,35,36,43], and influenced by structural and numerical chromosomal anomalies, as in cancer cells [42].

It is becoming increasingly clear that patterns and mechanisms of nuclear lamin association with the genome are more complex and dynamic than previously understood. Functional and live-cell imaging studies are needed in order to fully understand the mechanisms by which lamins contribute to organizing the 3D conformation of chromatin in the nucleus.

## Materials and methods

### Cell culture

HepG2 cells were cultured in DMEM/F12 (Life Technologies) with 10% fetal calf serum and 1% penicillin-streptomycin. Cells were also cultured with 10 µM CsA (Novartis; diluted 1:1000 from a 10 mM stock in cell media) for 72 h before harvest.

### Plasmids and transfections

HepG2 cells were transiently transfected with pCMV-Flag-preLA-WT (lamin A wt) and pCMV-Flag-preLA-L647R (pre-lamin A L647R) plasmids [27] using Lipofectamine 2000 (ThermoFisher). Lamin A expression was assessed by immunostaining using anti-Flag antibodies. Cells were analyzed by immuno-FISH after 48 h using anti-Flag antibodies and indicated FISH probes.

### Immunoblotting

Proteins were separated by 10% SDS-PAGE, transferred onto an Immobilon-FL membrane (Millipore) and membranes blocked with Odyssey blocking buffer (LI-COR). Membranes were incubated in Odyssey blocking buffer with antibodies against lamin A/C (1:2000, sc7292x, Santa Cruz Biotechnology; or a rabbit anti-lamin A/C antibody [44]; 1:1000), pre-lamin A (1:200, sc6214, Santa Cruz Biotechnology), lamin B1 (1:1000, sc6216, Santa Cruz Biotechnology), ZMPSTE24 (1:1000, AP2415a, Abgent) or γ-tubulin (1:10,000, T5326, Sigma-Aldrich). Proteins were detected with IRDye-800- or IRD IRDye-680-coupled secondary antibodies.

### Immunofluorescence

Cells cultured on coverslips were fixed with 3% paraformaldehyde and permeabilized for 10 min in PBS/0.1% Triton X-100. Cells were blocked with 2% BSA in PBS/0.1% Tween (PBST-BSA) for 30 min and incubated with primary antibodies in PBST-BSA overnight at 4°C. Cells were incubated with secondary antibodies in PBST-BSA for 45 min and washed in PBST-BSA before mounting with DAPI (1:1000, D-9942, Sigma-Aldrich). Antibodies used were mouse anti-lamin A/C (1:1000, sc7292x, Santa Cruz Biotechnology), goat anti-pre-lamin A (1:100, sc6214, Santa Cruz Biotechnology), anti-mouse Alexa Fluor® 594 and anti-goat Alexa Fluor® 488 (both 1:1000, Jackson ImmunoResearch).

### FISH

FISH was done as described [27]. Cells were incubated in 0.25% KCl/0.5% tri-sodium citrate for 10 min, fixed in ice-cold 3:1 methanol:acetic acid and dropped on slides. BAC FISH probe DNA (BacPac Resource Center) was labeled using Biotin-16-dUTP (Roche). 200–300 ng labeled probe per slide was mixed with 8 µg Cot-1 DNA and 30 µg salmon sperm DNA (Invitrogen) and precipitated. DNA was dissolved in 11 µl hybridization mix (50% deionized formamide, 2x SSC, 1% Tween 20, 10% dextran sulphate) at 42°C for 20 min and pre-annealed for 1 h at 37°C. Slides were RNase-treated and washed twice in 2x SSC, dehydrated in ethanol and air-dried. Slides were denatured for 1 min 20 sec in 70% deionized formamide/2x SSC (pH 7.5) at 70°C, dehydrated in ice-cold 70%, 90% and 100% ethanol and air-dried. Probes were denatured for 5 min at 70°C and pre-annealed for 15 min at 37°C. 10 µl of probe was applied onto coverslips which were then mounted on a slide. Slides were hybridized overnight at 37°C. Slides were washed 4x in 2x SSC at 45°C and 4x in 0.1x SSC at 60°C. Slides were blocked in 5% skim milk and incubated at 37^o^C with Avidin-Alexa Fluor 488 (Invitrogen; 1.7 µg/ml). Slides were washed in 4x SSC/0.1% Tween 20 and incubated with Biotinylated Anti-Avidin D (goat; 1.0 µg/ml; Vector) for 45 min at 37°C. Slides were washed and incubated with Avidin-Alexa Fluor 488 as above and mounted with DAPI in Dako Fluorescent Mounting Medium.

Based on ChIP-seq profiles, FISH probes were designed for regions changing LAD class after CsA (Supplementary Table S5). Since HepG2 cells harbor copy number variations, probes were designed outside these areas. 100 FISH images (200 alleles) were analyzed using FISHfinder [45] to calculate the distance to the nuclear periphery determined by the edge of DAPI staining. Distance to the periphery was normalized to the radius of the nucleus. T-tests were used to determine the significance of distance differences between control and CsA-treated cells.

### Immuno-FISH

Immuno-FISH was done as described [46]. Transfected cells grown on slides were fixed with 3% paraformaldehyde for 15 min, washed 3 × 5 min in PBS, blocked, and permeabilized in PBS with 0.01% Tween-20, 2% BSA, and 0.1% Triton X-100 for 30 min. Cells were incubated for 2 h with anti-Flag antibodies (1:1000, F1804, Sigma-Aldrich) and for 45 min with mouse anti-FLAG and rabbit anti-mouse Alexa 594 antibodies (1:1000, Jackson ImmunoResearch). Slides were washed 3 × 5 min in PBS with 0.01% Tween-20 and 2% BSA between and after incubations. Immunostaining was fixed with 3% PFA for 15 min. Slides were washed 3× in PBS, 2 × 2 min in 2× SSC and 3 min in 2× SSC at 80°C. Slides were denatured for 20 min in 80°C in 70% deionized formamide (Ambion) in 2× SSC (pH 7.5). FISH probes were labeled and ensuing FISH procedure was done as described above.

### ChIP-sequencing

ChIP was done as described [14]. Cells (5×10^6^ cells per ChIP) were cross-linked with 1% formaldehyde and lysed in RIPA buffer (140 mM NaCl, 10 mM Tris-HCl, pH 8.0, 1 mM EDTA, 0.5 mM EGTA, 1% Triton X-100, 1% SDS, 0.1% sodium deoxycholate, 1 mM PMSF, protease inhibitors). To generate 200–500 bp DNA fragments, cells were sonicated 4 × 10 min in a Bioruptor (Diagenode). After sedimentation, the supernatant was diluted 10x in RIPA and incubated with anti-lamin A/C (10 µg, sc7292x, Santa Cruz Biotechnology) or lamin B1 (10 µg, ab16048, Abcam) antibodies coupled to Dynabeads Protein G (Invitrogen). ChIP samples were washed 3x in ice-cold-RIPA. Samples were incubated for 6 h at 68°C in elution buffer (50 mM NaCl, 20 mM Tris-HCl, pH 7.5, 5 mM EDTA, 1% SDS) and 40 ng proteinase K for crosslink reversal and DNA elution. DNA was extracted and libraries prepared and sequenced on an Illumina HiSeq2500.

### ChIP-seq data processing

ENCODE ChIP input data for HepG2 cells were downloaded from NCBI GEO accession No. GSE29611. Lamin A and B ChIP-seq reads and input reads were mapped to hg19 using Bowtie v2.25.0 [47] with default parameters after removing duplicate reads using Picard’s MarkDuplicates. To avoid normalization bias, we ensured that each pair of aligned input and ChIP read files had the same read depth by down-sampling reads for each chromosome individually. Mapped reads were used to call LADs using Enriched Domain Detector (EDD) [11] with the following alteration. To account for technical variation that might occur in LAD calling, we first ran EDD 10 times on each lamin ChIP-seq dataset in auto-estimation mode for GapPenalty (GP) and BinSize (BS). Average GP standard deviation from the 10 runs was < 1 unit, while BS did not vary. GP variations elicited minimal alterations in LAD calls, allowing estimation of technical variability. Median length of these variable subdomains was 0.39 Mb for all LADs, i.e. < 2% of total LAD coverage, and 10–20 times smaller than median A- and B-LAD sizes. We concluded that intrinsic EDD variability did not significantly impact LAD calling. Average GP and BS values were then used to manually set GP and BS before a final EDD run with each dataset. Parameters were, for control/CsA conditions, respectively: lamin A, BS 16/17 kilobase (kb), GP 4.1/3.9; lamin B, BS 16/16, GP 5.4/5.9. Intersects between LADs and genes were computed using BEDTools v2.21.0 [48] and BEDOPS v2.4.27 [49]. Scripts were written in Perl [50] or R [51] and ggplot2 in R was used for plots. Browser files were generated by calculating ratios of ChIP over input for each 1 kb bin with input normalized to the ratio of total ChIP reads over total input reads.

### 3D genome modeling using chrom3D

#### Hi-C data processing

Hi-C data of HepG2 cells were downloaded from ENCODE (GEO accession No. GSM2825569) [52] and processed using HiC-Pro [53] with default settings to generate Hi-C contact matrices. The pipeline creates intra- and inter-chromosomal contact matrices at various bin sizes. We chose a 50-kb bin size intra-chromosomal contact matrices and 1-Mb bin size inter-chromosomal contact matrices for analyses. 50-kb contact matrices were used to call TADs using Armatus v2.3 [54].

#### 3D models

We applied Chrom3D to generate 3D genome models from Hi-C and lamin B1 ChIP-seq data [27,28]. We generated 800 models each for control and CsA-treated cells, using Hi-C data for HepG2 cells [52] and the lamin B1 LADs mapped in this study for control and CsA-treated cells. In the simulations, chromosomes are modeled as beads on a string, each bead representing a TAD, and of size proportional to the linear size of the TAD. For each model we computed the Euclidean distance between beads and the nucleus center. We then extracted all beads (and their Euclidean distances) harboring lamin A or lamin B LADs and classified these LADs as A-only, B-only and A-B LADs (or beads) for statistical analyses (see below) and illustrations. In the models, distances between a bead and the nuclear center were normalized by dividing the Euclidian distance by the radius of the modeled nucleus (5 µm).

### RNA-seq and gene expression analysis

Total RNA was isolated from two CsA exposure experiments using the RNeasy Mini Kit (Qiagen). Libraries were sequenced on an Illumina HiSeq2500. RNA-seq reads were processed with Tuxedo [55]. TopHat v2.10 was used to align reads with no mismatch against the hg19 reference genome with default settings, applying the Bowtie2 preset option ‘b2-very sensitive’ [56,57]. Transcript abundance was estimated using cufflinks v2.2.1, and differential gene expression determined using cuffdiff v2.2.1 [55]. Genes with absolute FPKM change < 0.05 between control and CsA conditions were considered stably expressed. A gene was ascribed to a LAD class if its transcription start site overlapped with the LAD.

## Statistical analyses

FPKM levels from RNA-seq data were compared using unpaired t-tests. For LAD size comparisons, a 2-way ANOVA was first applied to determine the global significance of CsA treatment vs. control. A Tukey honest significant difference (HSD) test was then applied to determine the significance of any difference between LAD classes. Comparisons of normalized LAD distances to the nuclear center in Chrom3D models and in FISH analyses were done using unpaired t-tests.

### Data access

Our ChIP-seq and RNA-seq data are available under GEO accession No. GSE119631. ChIP input data are available under GEO accession No. GSE29611. Hi-C data for HepG2 cells [52] are available under GEO accession No. GSM2825569.
