# Supplementary material for: Interplay of lamin A and lamin B LADs on the radial positioning of chromatin
Source: Nucleus. 2019 Jan 20;10(1):7–20. doi: 10.1080/19491034.2019.1570810 (PMC6363278; doi:10.1080/19491034.2019.1570810)
Supplement: Supplemental Material [file kncl-10-01-1570810-s001.pdf]

Supplementary information

Supplementary Figures

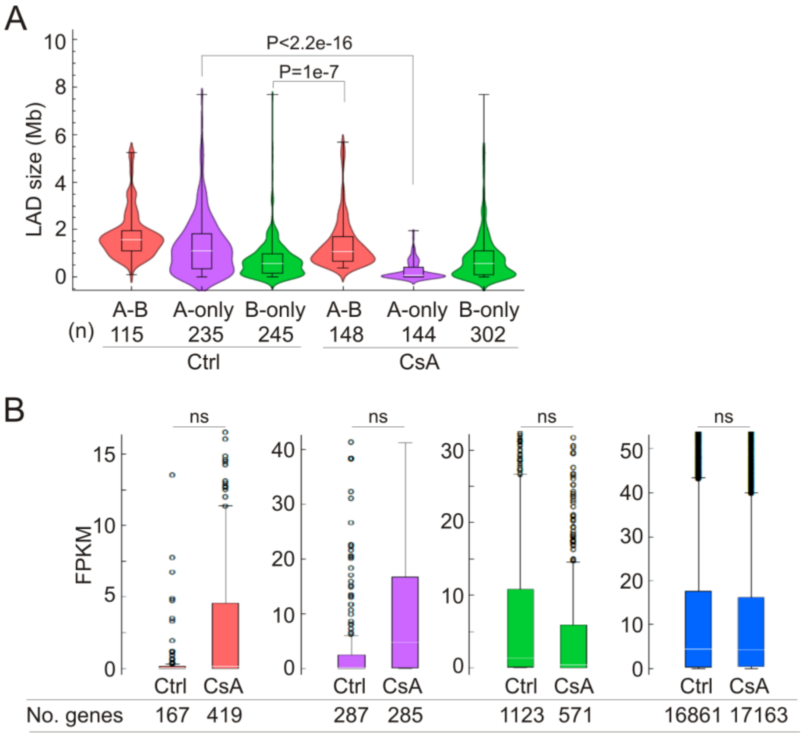

Figure S1. Characterization of LADs in control and CsA-treated HepG2 cells. (A) LAD size distribution, P-values shown only for significant differences (Tukey test after 2-way ANOVA.). (B) Gene expression level in each LAD class. No significant differences are detected (ns) at the P < 0.01 level (unpaired t-tests). FPKM values are from duplicate RNA-seq data.

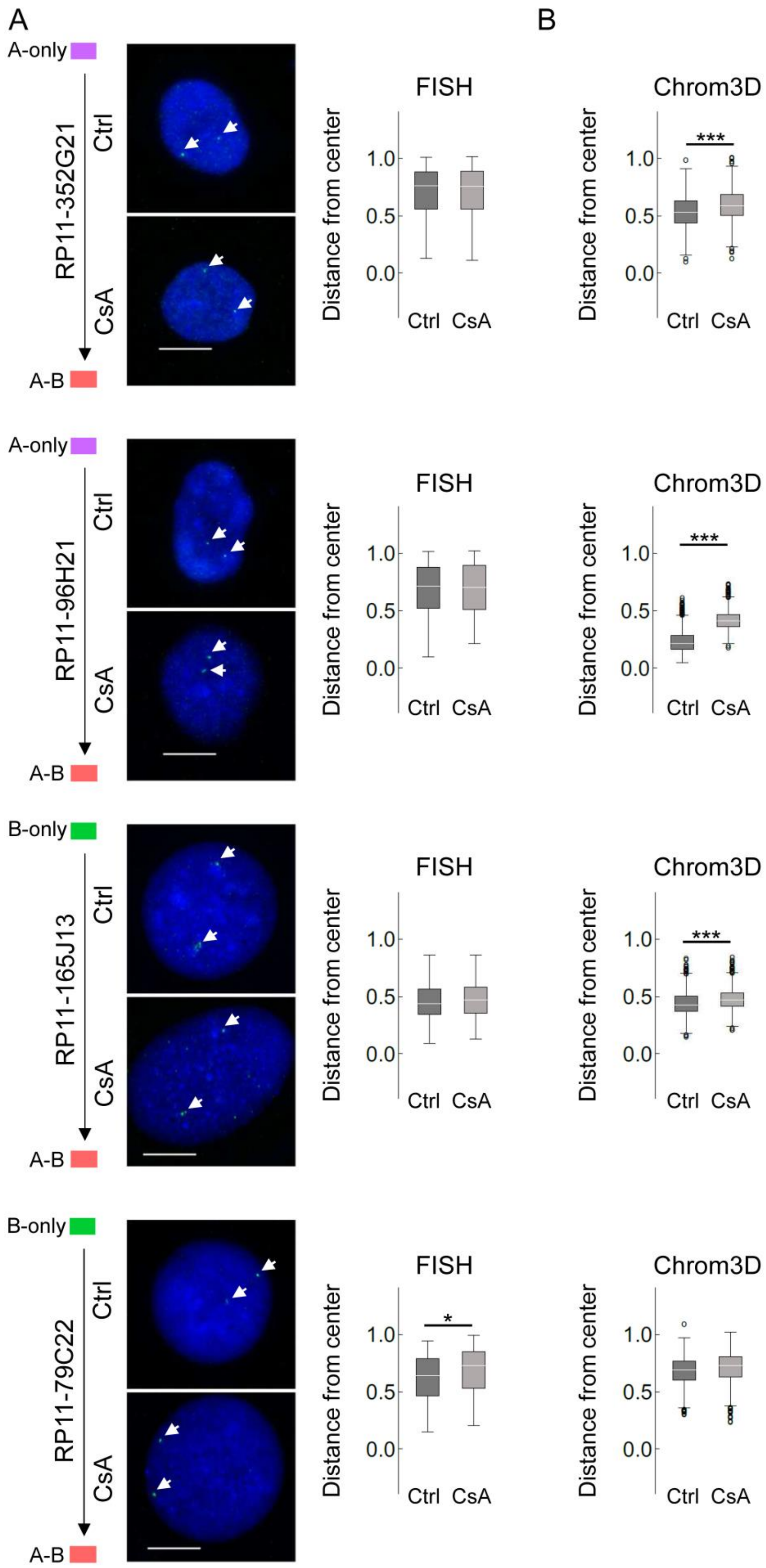

Figure S2. (part 1; legend on next page).

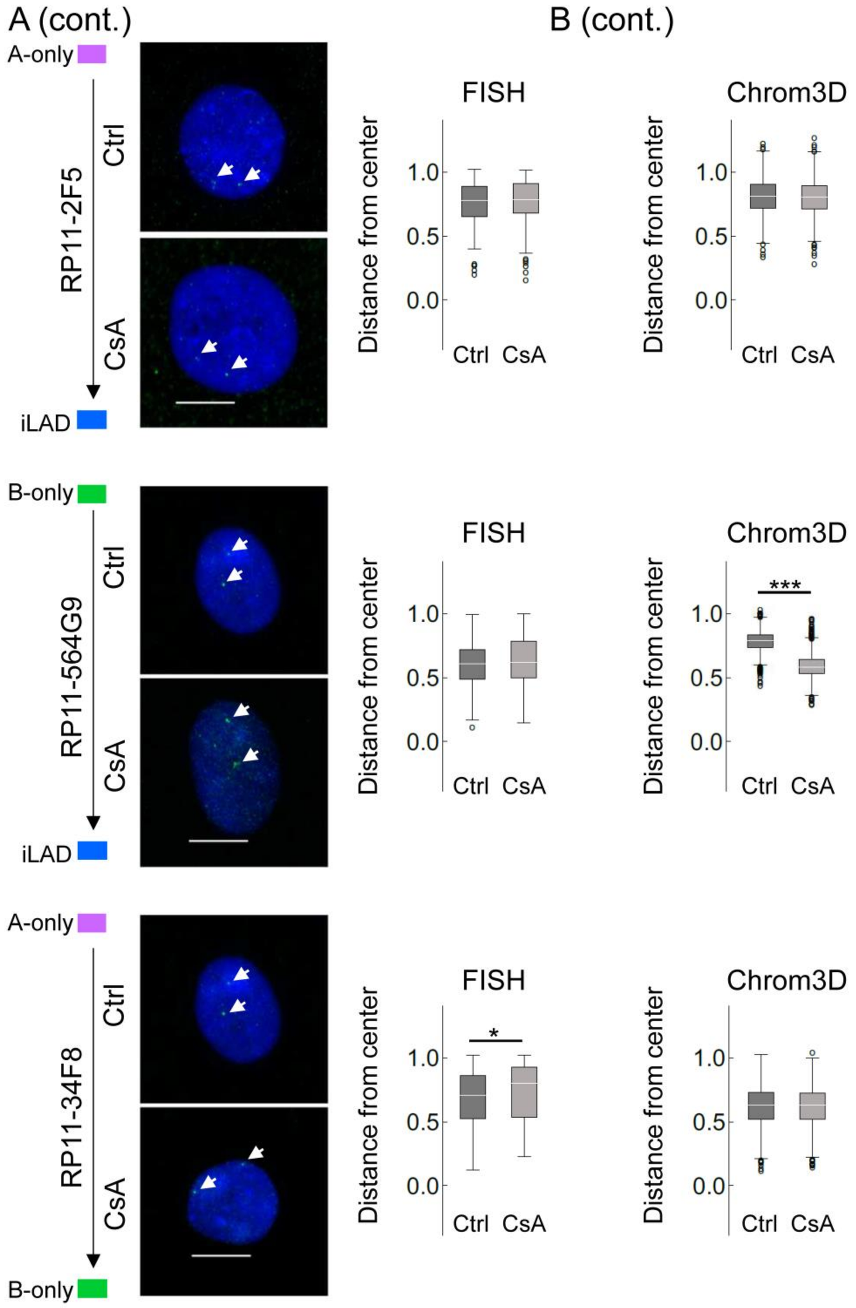

Figure S2. Chrom3D and FISH analysis of radial repositioning of loci after CsA treatment. (A) FISH analysis of locus positioning (arrows) in indicated LAD class in control and CsA-treated cells. Representative FISH images are shown, with normalized probe distance from the nucleus center (0 = center; 1 = periphery defined as the border of DAPI staining); n = 200 FISH signals analyzed per probe per condition; bars, 10 µm. (B) Normalized FISH probe distance from the nucleus center measured in 800 Chrom3D models. Probe number is shown. FISH: *P = 0.006, unpaired t-tests; Chrom3D: ***P < 2.2×10^{-16}, unpaired t-tests.

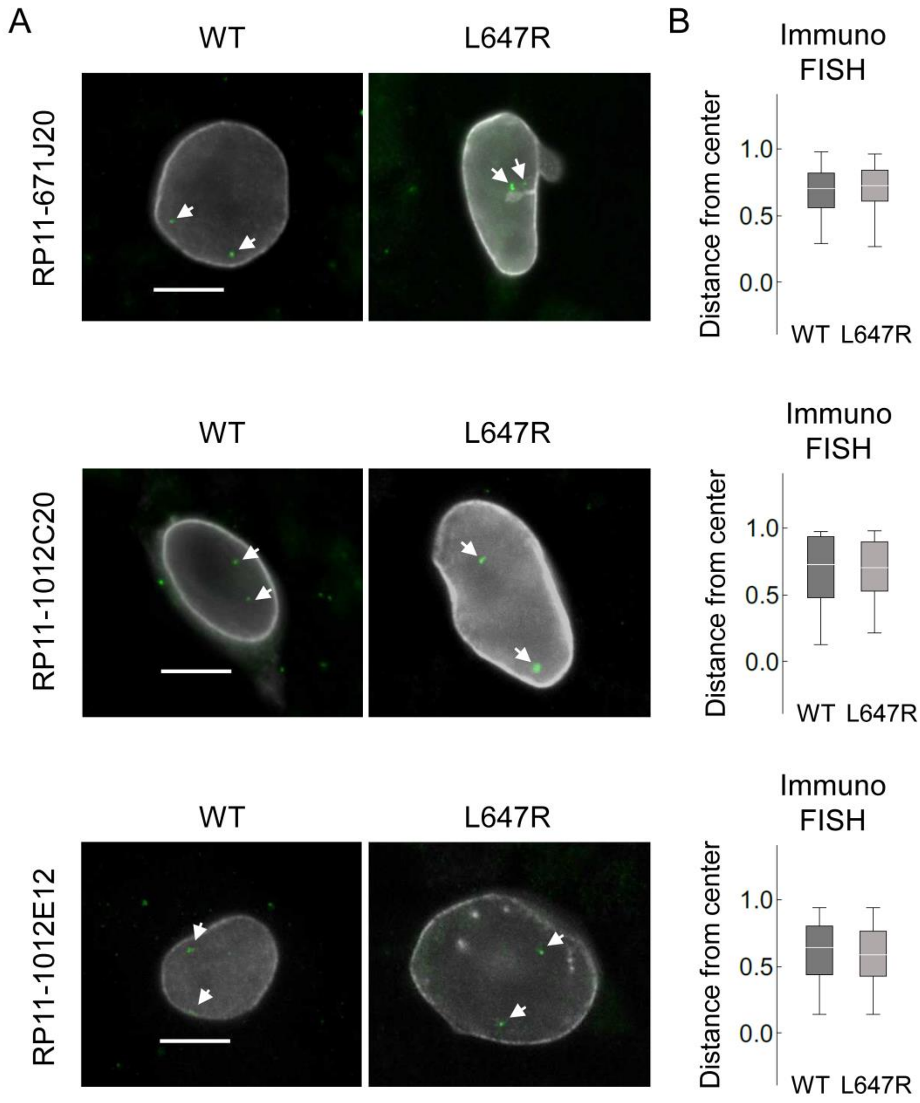

Figure S3. Expression of pre-lamin A wild type or pre-lamin A (L647R) in HepG2 cells does not radially relocalize loci. (A) Immuno-FISH images of positioning of indicated loci (detected by FISH probes; arrows) in HepG2 cells expressing either Flag-tagged wild-type lamin A (WT) or a Flag-tagged lamin A L647R mutant. Lamin A proteins were detected using anti-Flag antibodies. Probe number is shown; bars, 10 µm. (B) Quantification of relative distances of FISH signals from the nucleus center (0 = center; 1 = periphery defined by the border of Flag-lamin A staining at the nucleus edge); n = ~100 nuclei per sample. Distances are not significantly different between the two cell types ($P > 0.05$; unpaired t-tests.).

Supplementary Tables

Table S1. Gene expression level (FPKM) and LAD class in control and CsA-treated cells. (Excel)

Table S2. Lamin A and lamin B LAD characteristics in control and CsA-treated cells.

Table S3. Characteristics of LAD classes and inter-LADs in control and CsA-treated cells.

Table S4. Characteristics of LAD class transitions.

Table S5. FISH probe information.

Table S2. Lamin A and lamin B LAD characteristics in control and CsA-treated cells.

	LAD class	No. LADs	Genome coverage (% mappable)	Coverage (Mb)	No. genes	Genes / Mb	Median (Mb)
**Ctrl**	A	244	17.8	510.05	1673	3.28	1.73
	B	239	13.7	392.53	2614	6.66	1.28
**CsA**	A	178	8.6	245.55	1535	6.25	1.16
	B	278	16.5	470.77	2484	5.28	1.25

Table S3. Characteristics of LAD classes and inter-LADs in control and CsA-treated cells.

	LAD class	No. LADs	Genome coverage (%)	Coverage (Mb)	No. genes	Genes / Mb	Median (Mb)
**Ctrl**	A-B	115	7.1	202.03	659	3.26	1.56
	A-only	235	10.8	308.02	1045	3.39	1.11
	B-only	245	6.7	190.5	2033	10.67	0.59
	Inter-LAD	642	75.5	2160.76	25633	11.86	1.24
**CsA**	A-B	148	7.3	208.32	1118	5.37	1.08
	A-only	144	1.3	37.23	427	11.47	0.07
	B-only	302	9.2	262.46	1435	5.47	0.58
	Inter-LAD	574	82.2	2353.31	26390	11.21	1.21

Table S4. Characteristics of LAD class transitions.

LAD class (Ctrl)	LAD class (CsA)	No. LADs	Genome coverage (%)	Genome coverage (Mb)	No. genes	Genes / Mb	Median (Mb)
A-B	A-B	80	4.4	127.03	430	3.38	1.59
	A-only	13	0.1	1.97	19	9.65	0.15
	B-only	79	2.4	68.78	192	2.79	0.87
	Inter-LAD	21	0.1	4.24	18	4.24	0.20
A-only	A-B	36	0.4	12.85	104	8.09	0.19
	A-only	32	0.2	5.64	40	7.09	0.06
	B-only	97	2.7	76.79	199	2.59	0.50
	Inter-LAD	206	7.4	212.74	702	3.30	0.70
B-only	A-B	106	2.1	60.35	524	8.68	0.49
	A-only	50	0.4	12.37	163	13.18	0.06
	B-only	166	2.8	81.22	819	10.08	0.18
	Inter-LAD	129	1.3	36.56	527	14.42	0.11
Inter-LAD	A-B	41	0.3	8.08	60	10.7	0.07
	A-only	92	0.6	17.25	205	13.8	0.10
	B-only	142	1.2	35.66	225	4.4	0.11
	Inter-LAD	676	73.4	2099.77	25143	9.7	1.15

Table S5. FISH probe information.

Probe ID	Clone ID	Change LAD class	Chr.	Start pos.	End pos.	No. genes
p1	RP11-2F5	A switch to Inter-LAD	Chr.2	185216500	185377594	*7*
p2	RP11-671J20	Inter-LAD	Chr.3	9722941	9900724	*51*
p3	RP11-1012C20	B switch to Inter-LAD	Chr.1	2301890	2502158	*95*
p4	RP11-564G9	B switch to Inter-LAD	Chr.5	176865042	177055093	*66*
P5	RP11-79C22	A gain on B	Chr.10	7304838	7461350	*28*
P6	RP11-165J13	A gain on B	Chr.17	77197376	77328294	
P7	RP11-102E12	A switch to B	Chr.18	4467862	4624130	*24*
P8	RP11-34F8	A switch to B	Chr.11	40922548	41092384	*2*
P9	RP11-96H21	B gain on A	Chr.21	31932883	32103229	*61*
P10	RP11-352G21	B gain on A	Chr.9	8000830	8162993	*6*
